# HIV screening among newly diagnosed TB patients: a cross sectional study in Lima, Peru

**DOI:** 10.1186/s12879-018-3037-5

**Published:** 2018-03-20

**Authors:** Suzanne Ramírez, Fernando Mejía, Marlene Rojas, Carlos Seas, Patrick Van der Stuyft, Eduardo Gotuzzo, Larissa Otero

**Affiliations:** 1 Facultad de Medicina Alberto Hurtado, Universidad Peruana Cayetano Heredia, Av. Honorio Delgado 430, San Martín de Porres, 31 Lima, Peru; 2 Instituto de Medicina Tropical Alexander von Humboldt, Universidad Peruana Cayetano Heredia, Av. Honorio Delgado 430, San Martín de Porres, 31 Lima, Peru; 3 Hospital Cayetano Heredia, Ministry of Health, Av. Honorio Delgado 262, San Martín de Porres, 31 Lima, Peru; 4 Ministry of Health, Av. Salaverry 801, Jesús María, 15072 Lima, Peru; 5 Department of Public Health, Ghent University, De Pintelaan 185, 9000 Ghent, Belgium

**Keywords:** Voluntary counseling testing, Tuberculosis, HIV, Peru

## Abstract

**Background:**

Since 2006, the Peruvian National TB program (NTP) recommends voluntary counseling and testing (VCT) for all tuberculosis (TB) patients. Responding to the differential burden of both diseases in Peru, TB is managed in peripheral health facilities while HIV is managed in referral centers. This study aims to determine the coverage of HIV screening among TB patients and the characteristics of persons not screened.

**Methods:**

From March 2010 to December 2011 we enrolled new smear-positive pulmonary TB adults in 34 health facilities in a district in Lima. NTP staff offered VCT to all TB patients. Patients with an HIV positive result were referred for confirmation tests and management. We interviewed patients to collect their demographic and clinical characteristics and registered if patients opted in or out of the screening.

**Results:**

Of the 1295 enrolled TB patients, nine had a known HIV diagnosis. Of the remaining, 76.1% (979) were screened for HIV. Among the 23.9% (307) not screened, 38.4% (118) opted out of the screening. TB patients at one of the health care facilities of the higher areas of the district (OR = 3.38, CI 95% 2.17–5.28 for the highest area and OR = 2.82, CI 95% 1.78–4.49 for the high area) as well as those reporting illegal drug consumption (OR = 1.65, CI 95% 1.15–2.37) were more likely not to be screened. Twenty-four were HIV positive (1.9% of all patients 1295, or 2.4% of those screened). Of 15 patients diagnosed with HIV during the TB episode, ten were enrolled in an HIV program. The median time between the result of the HIV screening and the first consultation at the HIV program was 82 days (IQR, 32–414). The median time between the result of the HIV screening and antiretroviral initiation was 148.5 days (IQR 32–500).

**Conclusions:**

An acceptable proportion of TB patients were screened for HIV in Lima. Referral systems of HIV positive patients should be strengthened for timely ART initiation.

## Background

In 2016, 1.3 million people died from tuberculosis (TB) and 1 million people died from HIV. Of the 10.4 million people that develop TB each year, one out of ten is HIV positive [1]. HIV is one of the most important challenges to TB control globally [2]. By severely affecting the immune system, HIV facilitates TB dissemination and increases mortality of coinfected persons as compared to TB patients who are HIV negative [2, 3]. Active TB is an AIDS-defining disease and a criterion to start antiretroviral therapy (ART); therefore, diagnosis of both diseases is important in co-infected patients [4–8].

The optimal HIV screening strategy has been debated since the availability of HIV screening tests in 1985. Initially, HIV screening was mainly done in blood banks. In 1987, counseling and screening was recommended to persons at risk of HIV infection such as those with risk behaviors and those seeking care for sexually transmitted infections (STI). In 1993, screening was extended to patients attending hospitals that reported an HIV prevalence above 1% among their patients. In 1995, learning that zidovudine could prevent HIV vertical transmission prompted the implementation of universal screening among pregnant women [9]. Despite these efforts, a low proportion of persons infected with HIV were aware of their status, and a high proportion of patients were diagnosed in late stages of HIV infection. To increase HIV diagnosis, a voluntary counseling and testing strategy required scaling up [10]. In 2005, the Center for Disease Control (CDC), recommended provider-initiated counseling and testing to all persons between 13 and 64 years unless the person opts out [9, 11]. The World Health Organization (WHO) recommends HIV voluntary counseling and testing as a standard practice among TB patients to increase HIV status awareness [3]. However, in 2016, the WHO estimated that 57% of notified cases of TB were aware of their HIV status [1].

In Peru, HIV is concentrated among high-risk groups and 0.4% of the general population is infected, on the other hand, TB burden is among the highest in the region: in 2016 the incidence for all TB cases was 117 per 100,000 population [1]. As the burden of TB is higher, case detection and treatment is conducted in the first level of health care. Conversely, because of its low prevalence, HIV/AIDS is managed in referral centers [12]. TB nurses working in peripheral health facilities offer voluntary counseling and testing for HIV of TB patients routinely. Peru adopted the HIV voluntary counseling and testing strategy among TB patients in 2006 [13]. The WHO reported that of the 31,079 TB patients notified in 2016, 84% were screened for HIV. Of them, 6% were HIV positive [14]. TB-HIV co-infection is a challenge for public health, both for diagnosis and timely management and for the prognosis of both diseases. It is essential to expand HIV screening of TB patients and timely initiation of ART to improve individual prognosis, to reduce HIV transmission and to reduce the burden of HIV and TB. This study aims to determine the proportion of newly diagnosed TB patients screened for HIV in a district with high TB incidence in Lima, Peru and to analyze patient’s and health system’s characteristics associated to not being screened for HIV.

## Methods

### Study design and setting

We conducted a secondary data analysis of cross sectional data obtained within a cohort study in 34 health care facilities (one district hospital and 33 primary care health centers) managed by the Ministry of Health in the San Juan de Lurigancho district in Lima, Peru. This was a cohort study to determine the proportion of drug resistant TB and the rate of recurrent episodes among TB patients. A sample size of 1130 was calculated for those objectives. Adults with a smear-positive pulmonary TB episode diagnosed between March 2010 and December 2011 with no history of previous TB treatment were enrolled. Patients initiated anti-TB treatment at the TB clinic and were followed at the health facility until the end of their treatment regimen. Treatment outcomes were prospectively obtained from the NTP registers. TB registers were monitored monthly up to two years after the end of treatment of the last enrolled case for TB recurrence. If a recurrent episode was found among an enrolled and cured TB patient, a sputum sample was collected [15, 16]. San Juan de Lurigancho is a periurban district in the north east of Lima and lies on the side of a hill. It is the most populous district in Peru, with over one million inhabitants. In 2015, TB incidence in Lima was 164.9 per 100,000 inhabitants [17].

The NTP guidelines recommend that all TB patients should offered HIV screening with an ELISA or a rapid test after a pre test counseling session conducted by a TB nurse trained by the HIV program. A post test counseling session is given in referral health facilities where HIV multidisciplinary teams provide HIV care [18].

### Study participants

The cohort study included consenting adult patients with a first episode of smear positive pulmonary TB diagnosed between March 2010 and December 2011 in one of the study sites. Patients that had received more than two doses of TB treatment were excluded. This secondary data analysis included all cohort participants.

### Study procedures

Trained field workers enrolled participants and applied a structured questionnaire to collect demographic, epidemiological and clinical data. The variables sex, age, weight loss, education, social economical status, marital status, illegal drug consumption, alcohol abuse, history of deprivation of liberty, history of diabetes mellitus, employment and place of birth were obtained through face to face interviews while smear, culture and drug susceptibility test (DST) results, treatment outcomes, and HIV test results were retrieved from clinical files. A single sputum specimen was collected from each participant at enrollment. Sputa were transported to the Tuberculosis Laboratory of the Instituto de Medicina Tropical Alexander von Humboldt analyzed under smear microscopy and cultured on Löwenstein-Jensen media. The proportion methods evaluated drug susceptibility to isoniazid, rifampicin and ethambutol. Results were reported to the health facility [15, 16]. The Tuberculosis Laboratory at the Instituto de Medicina Tropical Alexander von Humboldt conducted regular quality control assurance procedures for smear microscopy, culture and DST.

Study participants that were not yet screened for HIV by the TB routine staff at the moment of the interview, were invited for screening. Those agreeing to be screened received counseling by the Ministry of Health designated staff and the study field workers drew a sample of blood. An ELISA or a rapid test was done. If positive, the diagnosis of HIV infection requires a confirmatory test (Indirect Immunofluorescence-IFI or Western Blot). ELISA for HIV was done at the Instituto de Medicina Tropical Alexander von Humboldt. Results were given to the TB staff that delivered the results to patients and managed them accordingly. Patients with a positive test (either obtained by the Ministry of Health routine procedures or by the study) were referred to HIV services (in Peru, these are located in referral hospitals) for a confirmatory test and post test counseling. If HIV was confirmed they were enrolled in the Ministry of Health HIV program where ART is provided free of cost to patients. The criteria to initiate ART [19] at the time of the study (2010–2011) were: Any symptomatic HIV-positive person with clinical stage B or C regardless CD4 count, any HIV-positive person with a CD4 count of 200 cells / mm3 regardless of clinical stage and other criteria determined by an expert committee. Clinical files of all TB-HIV positive patients were reviewed at the end of their TB treatment. We registered the date in which they were enrolled in the HIV program at the referral facility and the date of ART initiation.

For the cohort study logistics, the district was divided in four geographical areas depending on the altitude related to the hill of the study district and balancing the number and size of facilities in each area: highest, high, middle and lower. A field worker was assigned to each area. The two higher areas lie by the side of the hill that is less urbanized with poorer housing quality, and the middle area and lower areas, are on the flatter and more urban area of the district.

### Data management and analysis

Data were entered in a specially designed database in Access for Microsoft Office (Microsoft Corporation, Redmond, WA, US) and analyzed using Epi Info ™ version 7.1. The outcome was HIV screening (done or not done). Patients not screened were further categorized in “opted out” which included participants that explicitly opted out of HIV screening by saying “no, I do not want to be screened” and in “screening not done for unknown reasons” which included participants that did not explicitly opted out, but either HIV screening was not offered for unknown reasons or despite it being offered, it was not done for unknown reasons. This categorization aimed to compare the characteristics of patients opting-out to those who did not explicitly opted out but screening not done for diverse reasons (patient may not want to be screened but does not say so, or he/she may want but it is not done, because of lack of follow up by the staff or other reasons). The hypothesis underlying this subclassification was that TB patients opting out of HIV screening may have a higher risk of HIV infection and fear of it makes them opt out. While this hypothesis cannot be confirmed in this study, a comparison of patients opting out with those not screened without explicitly opting out, could be relevant. Age was categorized in young adults (18–34 years), adults (35–59 years) and older adults (60–90 years). The 22-months study period was divided in four periods to determine time trends in HIV screening. HIV screening could be different in time if there were screening tests stock outs, or if staff changes in time affected performance related to HIV screening. Socioeconomic status was measured by a Peruvian Ministry of Finance validated scale and categorized patients in poor (including extreme and not extreme poverty) and not poor [20]. Alcohol consumption was measured using the validated CAGE four-question screening test [21]. Those that had a high suspicion of alcoholism we classified as alcoholics.

We developed contingency tables to compare the independent variables with the outcome. Three bivariate analyses of independent variables and outcomes were done: being screened versus not being screened, which included all patients enrolled; refusing screening versus not being screening for unknown reasons, which included patients that were not screened; and HIV positive versus negative, which included all those screened. Odds ratios (OR) were calculated with 95% confidence intervals (CI). Two multivariate models were constructed using logistic regression, one for predictors of being screened for HIV, and one for predictors of opting out among those not screened for HIV. Both multivariate analyses were done in a stepwise backward manner. Variables associated with the outcome at a *p* < 0.2 significance as well as others relevant for each of the two outcomes were included in the multivariate model. We excluded missing data in the analysis of the determinants of not being screened for HIV. We included 1197 patients (98 were excluded for missing data: 88 on socio economic status and 10 for all other independent variables) in the multivariable model.

## Results

### Study population and HIV prevalence among TB patients

**Fig. 1:**
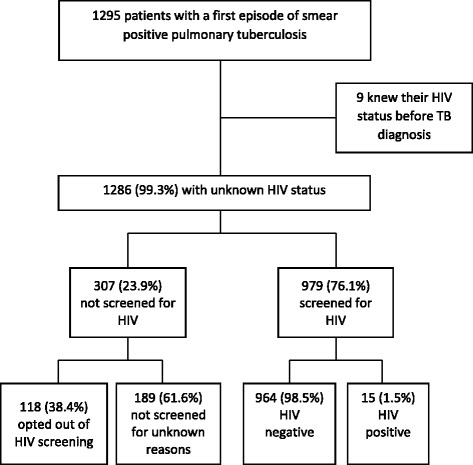
Patients with a first episode of tuberculosis and HIV screening in San Juan de Lurigancho, 2010–2011

The cohort study included 1295 patients and all were included in our analysis. Figure 1 describes the study population, those screened for HIV and the HIV screening results. Nine were aware of their HIV positive status before the TB diagnosis and fifteen were diagnosed with HIV during the TB episode. HIV prevalence was 1.9% (24/1295) among all TB patients, while it was 2.4% (24/988) among those with a known HIV status (excluding those not tested).

### HIV screening coverage and determinants of screening

Of all HIV screenings conducted among the study participants (76.1%, 979/1286): 81.2% (795/979) were done by the routine TB staff, 18.8% (184/979) were done by the study field workers. Therefore, if the study staff had not conducted additional efforts to those of the routine staff to screen TB patients for HIV, coverage of HIV screening among new TB patients would have been 61.8% (795/1286). Table 1 describes the characteristics of patients that were screened, of those opting out and of those not screened for unknown reasons.

**Table 1:** Characteristics of patients with a first episode of tuberculosis by their HIV screening status, San Juan de Lurigancho, 2010–2011

Characteristics	Screened	Not screened for unknown reasons	Opted out of screening
	*N* (%)	*N* (%)	*N* (%)
Sex			
Female	394 (39.9%)	69 (36.3%)	39 (33.1%)
Male	593 (60.1%)	121 (63.7%)	79 (66.9%)
Age, in years			
18–34	721 (73.0%)	137 (72.1%)	83 (71.0%)
35–49	210 (21.3%)	41 (21.6%)	24 (20.5%)
> 50	57 (5.7%)	12 (6.3%)	10 (8.5%)
Weight loss reported by the patient			
Yes	798 (81.1%)	162 (85.3%)	94 (79.7%)
No	186 (18.9%)	28 (14.7%)	24 (20.3%)
Location of health facility in district area			
Lowest area	199 (20.2%)	25 (13.2%)	8 (6.8%)
Middle area	314 (31.8%)	35 (18.4%)	12 (10.2%)
Highest area	259 (26.2%)	73 (38.4%)	63 (53.4%)
High area	215 (21.8%)	57 (30.0%)	35 (29.6%)
Education			
Primary school or less	400 (40.6%)	87 (45.8%)	60 (50.8%)
High school	365 (37.0%)	67 (35.3%)	36 (30.6%)
Higher education	221 (22.4%)	36 (18.9%)	22 (18.6%)
Socioeconomic status			
Poor	240 (26.0%)	48 (27.3%)	30 (28.3%)
Not poor	685 (74.0%)	128 (72.7%)	76 (71.7%)
Marital status			
Married / cohabiting	369 (37.4%)	65 (34.3%)	53 (45.0%)
Divorced	72 (7.3%)	16 (8.4%)	5 (4.2%)
Single	521 (52.8%)	101 (53.1%)	56 (47.5%)
Widow	25 (2.5%)	8 (4.2%)	4 (3.3%)
Illegal drug consumption			
No	838 (84.9%)	140 (73.7%)	99 (83.9%)
Yes	149 (15.1%)	50 (26.3%)	19 (16.1%)
Alcohol consumption (CAGE score)			
Alcoholism	52 (8.9%)	13 (10.7%)	7 (9.3%)
No alcoholism	534 (91.1%)	109 (89.3%)	68 (90.7%)
Ex prison inmate			
Yes	49 (5.0%)	8 (4.2%)	4 (3.4%)
No	936 (95.0%)	182 (95.8%)	114 (96.6%)
Diabetes mellitus, as reported by the patient			
Yes	41 (4.2%)	9 (4.7%)	5 (4.2%)
No	944 (95.8%)	181 (95.3%)	113 (95.8%)
Type of TB treatment regimen			
Regimen for drug sensitive TB	970 (98.3%)	189 (99.5%)	116 (98.3%)
Regimens for drug resistant TB	17 (1.7%)	1 (0.5%)	2 (1.7%)
Employment			
Yes	695 (70.4%)	138 (72.6%)	83 (70.3%)
No	190 (19.3%)	40 (21.1%)	21 (17.8%)
Student	102 (10.3%)	12 (6.3%)	14 (11.9%)
Study period			
1	261 (26.4%)	39 (20.5%)	34 (28.8%)
2	233 (23.6%)	42 (22.1%)	28 (23.7%)
3	258 (26.2%)	64 (33.7%)	18 (15.3%)
4	235 (23.8%)	45 (23.7%)	38 (32.2%)
Place of birth			
Coastal region	611 (62.0%)	116 (61.1%)	74 (62.7%)
Jungle region	138 (14.0%)	27 (14.2%)	19 (16.1%)
Andean region	237 (24.0%)	47 (24.7%)	25 (21.2%)

Among those screened for HIV, the median time between TB treatment initiation and HIV screening was 4 days (interquartile range 0–18 days). In 69.9% (684/979) patients, HIV screening was done before or within 15 days after TB treatment initiation -as per NTP guidelines- and after 15 days of starting TB treatment in 30.1% (295/979) patients.

Table 2 shows the determinants of not being screened for HIV of the 1197 patients included in the analysis (98 were excluded for missing data: 88 on socio economic status and 10 on a single other variable). Receiving TB care in one of the health care facilities of the higher areas of the district (odds ratio (OR) = 3.38, confidence interval (CI) 95% 2.17–5.28, *p* < 0.0001 for the highest area and OR = 2.82, CI 95% 1.78–4.49, *p* < 0001, for the high area) as well as reporting illicit drug consumption (OR = 1.65, CI 95% 1.15–2.37, *p* = 0.0062) was associated to not being screened for HIV.

**Table 2:** Determinants of not being screened for HIV among 1197 patients with a first episode of smear positive pulmonary tuberculosis, San Juan de Lurigancho, 2010–2011

Characteristics	Screened *N*(%)	Not screened *N*(%)	Crude OR (95% CI)	*p*	Adjusted OR (95%CI)	*p*
Sex						
Female	394 (78.5%)	108 (21.5%)	1			
Male	593 (74.8%)	200 (25.2%)	1.23 (0.94–1.63)	0.136		
Age, in years						
18–34	721 (76.6%)	220 (23.4%)	0.94 (0.68–1.31)	0.752		
35–49	210 (76.4%)	65 (23.6%)	1			
> 50	57 (72.1%)	22 (27.9%)	1.15 (0.63–2.09)			
Weight loss reported by the patient						
Yes	798 (75.7%)	256 (24.3%)	1.07 (0.76–1.51)	0.699		
No	186 (78.2%)	52 (21.8%)	1			
Location of health facility in district area						
Lowest area	199 (85.8%)	33 (14.2%)	1		1	
Middle area	314 (87.0%)	47 (13.0%)	0.89 (0.54–1.47)		0.90 (0.54–1.49)	< 0.001
Highest area	259 (65.6%)	136 (34.4%)	3.34 (2.15–5.21)		3.38 (2.17–5.28)	
High area	215 (70.0%)	92 (30.0%	2.8 (1.77–4.45)	< 0.001	2.82 (1.78–4.49)	
Education						
Primary or less	400 (73.1%)	147 (26.9%)	1.23 (0.91–1.66)	0.153		
High School	365 (78.0%)	103 (22.0%)	1			
Higher	221 (79.2%)	58 (20.8%)	0.88 (0.61–1.28)			
Socioeconomic status						
Poor	240 (75.5%)	78 (24.5%)	1.09 (0.81–1.48)	0.556		
Not poor	685 (77.0%)	204 (23%)	1			
Marital Status						
Married / cohabiting	369 (75.8%)	118 (2.2%)	1.12 (0.84–1.49)	0.523		
Divorced	72 (77.4%)	21 (22.6%)	0.88 (0.51–1.53)			
Single	521 (76.8%)	157 (23.2%)	1			
Widow	25 (67.6%)	12 (32.4%)	1.57 (0.75–3.29)			
Illegal drug consumption						
Yes	149 (68.3%)	69 (31.7%)	1.64 (1.16–2.32)	0.006	1.65 (1.15–2,37)	0.006
No	838 (77.8%)	239 (22.2%)	1		1	
Alcohol consumption (CAGE score)						
Yes	52 (72.2%)	20 (27.8%)	1.28 (0.74–2.22)	0.38		
No	534 (75.1%)	177 (24.9%)	1			
Ex prison inmate						
Yes	49 (80.3%)	12 (19.7%)	0.68 (0.31–1.47)	0.305		
No	936 (76.1%)	296 (23.9%)	1			
Diabetes Mellitus, as reported by the patient						
Yes	41 (74.5%)	14 (25.5%)	1.12 (0.60–2.08)	0.725		
No	944 (76.3%)	294 (23.7%)	1			
TB regimen						
Regimen for drug sensitive TB	970 (76.1%)	305 (23.9%)	1			
Regimens for drug resistant TB	17 (85.0%)	3 (15%)	0.70 (0.20–2.44)	0.556		
Employment						
Yes	695 (75.8%)	221 (24.2%)	1	0.646		
No	190 (75.7%)	61 (24.3%)	0.99 (0.71–1.40)			
Student	102 (79.7%)	26 (20.3%)	0.81 (0.50–1.28)			
Study Period						
1	261 (78.1%)	73 (21.9%)	1			
2	233 (76.9%)	70 (23.1%)	1.13 (0.76–1.67)	0.431		
3	258 (75.9%)	82 (24.1%)	1.18 (0.80–1.73)			
4	235 (74.0%)	83 (26.0%)	1.37 (0.94–2.01)			
Place of birth						
Coastal region	611 (76.3%)	190 (23.7%)	1	0.985		
Jungle region	138 (75.0%)	46 (25.0%)	0.97 (0.65–1.44)			
Andean region	237 (76.7%)	72 (23.3%)	0.98 (0.71–1.35)			

Table 3 compares the characteristics of patients opting out of HIV screening to those of patients not screened for unknown reasons. Attending a health care facility in the highest area of the district was associated to opting out of screening. Patients reporting illegal drug consumption and those enrolled in the third period of the study were more likely not to be screened because of unknown reasons as compared to opting out of screening.

**Table 3:** Determinants of opting out of HIV screening as compared to not being screened for unknown reasons in 307 smear positive tuberculosis patients not screened for HIV, San Juan de Lurigancho 2010–2011

Characteristics	Opted out of screening *N* (%)	Not screened for unknown reasons *N* (%)	Crude OR (95% CI)	*p*	Adjusted OR (95%CI)	*p*
Sex						
Female	39 (36.2%)	69 (63.8%)	1			
Male	79 (39.5%)	121 (60.5%)	1.14 (0.70–1.85)	0.595		
Age, in years						
18–34	83 (37.7%)	137 (62.27%)	1.04 (0.58–1.84)	0761		
35–49	24 (36.9%)	41(63.1%)	1			
> 50	10 (45.5%)	12 (54.5%)	1.42 (0.53–3.79)			
Weight loss reported by the patient						
Yes	94 (36.7%)	162 (63.3%)	0.67 (0.37–1.22)	0.194		
No	24 (46,2%)	28 (53.8%)	1			
Location of health facility in district area						
Lowest area	8 (24.2%)	25 (75.8%)	1		1	
Middle area	12 (25.5%)	35 (74.5%)	1.07 (0.38–3.00)	0.022	1.21 (0.42–3.49)	
Highest area	63 (46.3%)	73 (53.7%)	2.65 (1.12–6.30)		3.28 (1.32–8.18)	
High area	35 (38.0%)	57 (62.0%)	1.92 (0.78–4.72)		2.18 (0.86–5.56)	
Education						
Primary or less	60 (40.8%)	87 (59.2%)	1.28 (0.76–2.16)	0.627		
High School	36 (35%)	67 (65.0%)	1			
Higher	22 (37.9%)	36 (62.1%)	1.09 (0.55–2.13)			
Marital Status						
Married / cohabiting	53 (44.9%)	65 (55.1%)	1.44 (0.88–2.35)	0.219		
Divorced	5 (23.8%)	16 (76.2%)	0.56 (0.20–1.62)			
Single	56 (35.7%)	101 (64.3%)	1			
Widow	4 (33.3%)	8 (66.7%)	0.90 (0.26–3.13)			
Illegal drug consumption						
Yes	19 (27.5%)	50 (72.5%)	0.54 (0.30–0.98)	0.037	0.43 (0.23–0.80)	
No	99 (41.4%)	140 (58.6%)	1			
Alcohol consumption (CAGE score)						
Yes	7 (35.0%)	13 (65.0%)	0.74 (0.29–1.88)	0.524		
No	68 (38.4%)	109 (61.6%)	1			
Ex prison inmate						
Yes	4 (46.7%)	8 (53.3%)	0.81 (0.24–2.74)	0.726		
No	114 (38.5%)	182 (61.5%)	1			
Diabetes Mellitus, as reports by the patient						
Yes	5 (35.7%)	9 (64.3%)	0.90 (0.29–2.75)	0.849		
No	113 (38.4%)	181 (61.6%)	1			
TB regimen						
Regimen for drug sensitive TB	116 (38.0%)	189 (62.0%)	1			
Regimens for drug resistant TB	2 (66.7%)	1 (33.3%)	3.29 (0.29–36.6)	0.315		
Employment						
Yes	83 (37.6%)	138 (62.4%)	1			
No	21 (34.4%)	40 (65.6%)	0.88 (0.49–1.60)	0.217		
Student	14 (53.8%)	12 (46.2%)	1.96 (0.87–4.45)			
Study Period						
1	34 (46.6%)	39 (53.4%)	1		1	
2	28 (40.0%)	42 (60.0%)	0.79 (0.40–1.53)	0.003	0.65 (0.33–1.31)	
3	18 (22.0%)	64 (78.0%)	0.33 (0.17–0.67)		0.23 (0.11–0.49)	
4	38 (45.8%)	45 (54.2%)	0.99 (0.53–1.88)		0.71 (0.35–1.40)	
Place of birth						
Coastal region	74 (39.0%)	116 (61.0%)	1			
Jungle region	19 (41.3%)	27 (58.7%)	1.10 (0.57–2.12)	0.663		
Andean region	25 (34.7%)	47 (65.3%)	0.80 (0.45–1.42)			

### Determinants and management of HIV positive TB patients

In a bivariate analysis of the characteristics of HIV positivity (including both those diagnosed before the TB episode and those diagnosed during the TB episode) among the 988 patients screened, more men than women were HIV positive (18 (3.0%) vs. 6 (1.5%)), more adults than young adults (9 (4.3%) vs. and 15 (2.0%)) and more patients reporting illegal drug consumption were HIV positive than those reporting never having used illegal drugs (crude OR: 4.24, 95%CI 1.85–9.73). We had insufficient power to conduct a multivariate analysis of predictors of HIV positive status.

Of the nine patients known to be HIV positive before the TB episode, eight were already on ART. Of the 15 patients diagnosed with HIV during the TB episode, we found evidence that 10 were affiliated to the Ministry of Health HIV program at a referral hospital and 9 were started on ART. We did not find evidence of HIV program enrollment in five patients. These patients may have been enrolled in an HIV program without it being registered in their TB clinical files, they may have attended a private HIV care facility where they have to pay for care and ART, or they may have not been enrolled in an HIV program. The median time between the result of the HIV screening and the first medical consultation of the HIV program, among those 10 patients, was 82 days (IQR, 32–414). The median time between the result of the HIV screening and ART initiation was 148.5 days (IQR, 32–500). The median CD4 cell count among patients enrolled in HIV care during the TB episode was 189 cells/mm^3^ (IQR, 55–312).

## Discussion

We found an HIV screening coverage of 76.1% among patients recently diagnosed with smear positive pulmonary TB in an urban context of median TB incidence and low HIV prevalence. Factors associated to not being screened were illegal drug consumption and geographical location of the TB health facility within the study district. Fifteen TB patients were diagnosed with HIV during the TB episode, of which five did not have evidence of HIV program enrollment upon completion of TB treatment.

In the Americas region, in 2016, 80% persons notified for TB had a documented HIV positive test result, which is above the global proportion for 2016 (57%). In the same year, HIV screening in Peru covered 84% of TB patients [14]. In 2010 (when our study was conducted) the National TB Program reported that 76% of TB patients were screened for HIV [22]. In our study, conducted between 2010 and 2011, we found a fairly high coverage, however, the study staff did additional efforts to screen those that had not being initially screened.

Only 9.2% TB patients opted out of screening, while 14.7% were not screened for unknown reasons. Patients not screened without explicitly opting out may have not wanted to be screened but did not say so, or the screening may have been delayed or postponed for diverse reasons (patient’s reasons such as not being sure, or fearing HIV screening, or health system reasons such as inadequate follow up of screening request). In South Africa, voluntary counseling and testing was adopted in 2007 but less than half of the TB patient’s accepted HIV screening [23]. In India, 60% of TB patients were screened for HIV and 33% of new HIV diagnoses would have been missed if screening during the TB episode were not done [24].

Our finding that living in the higher areas of the district that lie by the hill as opposed to living in the middle and lower areas that are more urbanized, was associated to not being screened. Also, those living in the highest area were more likely to opt out of screening. This may be due to the fact that in higher areas of the district there are fewer health centers and road access may be more difficult. This area of the district has lower socioeconomic status, however, the association we found was independent of that factor and of level of education of the patient. This suggests that health care facility-related factors not measured in this study could be associated to screening coverage. Management of health facilities in Lima is organized in micronetworks within districts. There may be characteristics of these micronetworks in the higher area that do not favor screening, such as number of health staff dedicated to TB activities and poorer supervision practices. This should be further explored in future studies.

The single patient related factor –among those measured by the study- associated to not being screened was illegal drug consumption. The screening strategy in this group (17% of the TB patients in our study reported use of illegal drugs) should be reinforced. The use of any illegal drug is associated with risk behaviors that may increase the likelihood of acquiring or transmitting HIV infection. In South Africa, fear, low perception of risk and the wish of being treated first and only for TB as well as not being offered screening, was associated to not been screened for HIV [25]. In a similar study in India, uptake of HIV testing was significantly lower in older age groups and females [26]. In Cambodia, married patients, those with a previous HIV screening, a higher level of education and with more access to a health facility were more likely to be screened while self-perceived stigma was associated to not being screened [27]. Peru is a country with a median TB incidence and a concentrated HIV epidemic, but co-infection has increased in recent years. Despite the fact that since 2013, the NTP recommends that every patient diagnosed with TB have to be screened for HIV, 100% screening coverage has not yet been reached.

Our findings suggest the need of strengthening the reference systems between TB and HIV programs. Despite having a small number of HIV positive patients, we detected a delay between HIV positive result and HIV program enrollment and ART initiation, and we did not find evidence of HIV program enrollment in five patients. The HIV and TB program in Peru are partially integrated but care for both infections is provided in different facilities. Obtaining an appointment at referral hospitals in Lima, where HIV diagnosis is confirmed and managed, can take long and that could be contributing to the delays found [28]. Stigma has been cited in other settings as a cause that discouraged rapid linkage to care of recently diagnosed HIV-positive individuals [29]. Prompt linkage to care for early ART initiation is important to achieve rapid virologic suppression and reduce mortality [30, 31]. A better integration of both programs could reduce barriers [32–34] for timely care and facilitate the initiation of ART. Decentralizing HIV care to peripheral health facilities where TB is managed may facilitate geographical access. Yet, if peripheral facilities are located at the patient’s neighborhoods it may increase stigma and fear of disclosure of HIV diagnosis.

This operational study has some limitations. We did not obtain the exact reasons for why patients refused screening or why it was not done among those not opting out, which impedes a more precise explanation to our findings. Furthermore, we do not know which would have been the coverage if the study were not conducted. As we made additional efforts to those of the routine staff to screen patients for HIV, we probably overestimated the routine coverage. However, the coverage reported by this study, reflects the potential reach of HIV screening if it is systematically offered to all TB patients. We did not collect risk factors for HIV infection. If we had collected risk factors for HIV infection, we could compare the presence of risk factors among the patients screened, those that opted out and those that were not screened for unknown reasons. Opting out of HIV screening could result from a very low perception of risk or form fear of a positive result because of risk behavior. In studies in Ethiopia and South Africa, adults with TB thought that voluntary counseling and testing could delay TB treatment and that TB treatment should be prioritized [35]. Finally, the small number of HIV TB co-infected patients did not allow a multivariate analysis to predict HIV infection among TB patients. Among the strengths of the study was that it included a large number of TB patients representative of the TB population in the district.

## Conclusions

We found that 76.1% patients recently diagnosed with TB, were screened for HIV. Patients that reported illegal drug use were less likely to be screened. Patients with a recent HIV diagnosis took long to be enrolled in a program for the initiation of antiretroviral treatment. This suggests a suboptimal integration of TB and HIV services since TB is managed in primary care centers and HIV is managed in referral centers such as hospitals. Most studies on HIV screening among TB patients are from countries with high HIV prevalence. HIV screening and management among TB patients in settings with low HIV prevalence (less than 1%), such as Peru should be enhanced to reduce mortality from co-infection.

## Abbreviations

ART: antiretrovirals
CI: Confidence Interval
HIV: Human Immunodeficiency Virus
NTP: National Tuberculosis Program
TB: Tuberculosis
